# Effect of Physician Peer Coaching (PPC) on Burnout in Hospital-Based Physicians: A Pilot Randomized Controlled Trial

**DOI:** 10.7759/cureus.117780

**Published:** 2026-10-11

**Authors:** Prasanna Kumar Reddy Tadi, Guha J Ganesh, Sai Varun Bethina, Amanda J Young, Kodanda Ramulu Shalini Valusa, Brandon Eilberg, Pranav Chintkuntlawar, Jessica L Webster, Gregory A Brown

**Affiliations:** 1 Neurology, Bon Secours Health System, Richmond, USA; 2 Neurology, Geisinger Health System, Wilkes-Barre, USA; 3 Neurology, University of Pittsburgh Medical Center, Pittsburgh, USA; 4 Neurology, West Virginia University School of Medicine, Morgantown, USA; 5 Biostatistics, Geisinger Health System, Danville, USA; 6 Family Medicine, University of North Carolina Health, Chapel Hill, USA; 7 Emergency Medicine, Crozer Health, Upland, USA; 8 Bioinformatics, Virginia Commonwealth University, Richmond, USA; 9 Investigator Initiated Research Operations, Geisinger Health System, Danville, USA; 10 Orthopaedic Surgery, Adventist Health, Tillamook, USA

**Keywords:** emotional exhaustion, health wellness and promotion, peer coaching, physician burnout, physician training, professional burnout, stress & burnout

## Abstract

Introduction

Physician burnout (PB) is a significant and growing problem among hospital-based physicians. Coaching delivered by external professional coaches has previously been used to reduce burnout, but coaching delivered by trained physician peers has not been evaluated. This pilot randomized controlled trial examined whether physician peer coaching (PPC) is associated with lower burnout and a more favorable work environment for hospital-based physicians.

Methods

We conducted a pilot randomized controlled trial at Geisinger Health System, Wilkes-Barre, Pennsylvania, to assess whether PPC is associated with lower PB and more favorable work environment factors among hospital-based physicians (HBP). Fifty-one physicians were consented and randomized; 45 (23 intervention, 22 control) received the allocated condition and were included in the analysis (modified intention-to-treat). Twenty-three physicians in the intervention group (IG) received six peer-coaching sessions between June and December 2022 at three- to five-week intervals by a trained physician coach; the control group received no coaching until after the six-month assessment. Burnout was measured with the Maslach Burnout Inventory (MBI), and work environment with the Areas of Worklife Survey (AWS), at baseline, three, six, and nine months.

Results

In the between-group difference-in-differences analysis at six months, coaching was associated with a significantly greater reduction in emotional exhaustion (EE) (primary outcome −6.51, 95% CI −12.70 to −0.31; p=0.04), and a greater improvement in workload congruence [0.57, 95% confidence interval (CI) 0.13-1.01; p=0.01] relative to control. No other between-group difference reached significance, including overall burnout (OB) [odds ratio (OR) 0.58, 95% CI 0.17-2.05; p=0.40]. Exploratory within-group analyses: MBI repeated measures trends for IG (median scores at baseline, three, six, and nine months): EE (30, 25, 18, 17; p<.01); depersonalization (DP) (6, 5, 2.5, 3; p=0.01); personal accomplishment (PA) (37, 40, 41.5, 39; p=0.02); OB (60.9%, 52.2%, 40.9%, 33.3%; p=0.05); high EE (60.9%, 39.1%, 31.8%, 23.8%; p=0.01); high DP (30.4%, 30.4%, 22.7%, 14.3 %; p=0.18). AWS repeated measures trends for IG (median scores at baseline, three, six, and nine months); workload (2.2, 2.4, 2.4, 2.9; p<0.01); control (3.0, 3.3, 3.4, 3.0; p=0.49); reward (3.0, 3.5, 3.5, 3.5; p<0.01); community (3.4, 3.6, 3.7, 3.6; p=0.31); fairness (2.8, 3.0, 3.2, 3.0; p=0.21); values (3.5, 3.5, 3.9, 3.6; p=0.62). In the control group, OB did not significantly change (59.1% at baseline to 52.4% at 6 months; p=0.66; trend p=0.65), and no other burnout or work-environment measure showed a significant trend.

Conclusions and relevance

This pilot randomized controlled trial suggests that PPC may reduce EE and improve perceived workload among hospital-based physicians. Favorable within-group trends in DP, PA, OB, and reward were not confirmed by between-group comparison and should be considered exploratory. Within-group improvements persisted three months after coaching ended, but this observation is uncontrolled because the control group was offered coaching after six months. Confirmation in larger, adequately powered, multi-site trials is needed.

Trial registration

ClinicalTrials.gov identifier - NCT05251675.

## Introduction

Physician burnout is a prevalent and consequential occupational condition; the National Academy of Medicine has identified clinician burnout as a systems-level problem warranting coordinated action across health care organizations [1]. Burnout is seen across all specialties, with prevalence estimates ranging from 35%-54% [1]. A recent American Medical Association (AMA) news wire reported that emergency medicine (62%) and hospital medicine (59%) had the highest rates of burnout [2]. Physician burnout is a long-term stress reaction marked by emotional exhaustion (EE), depersonalization (DP), and a diminished sense of personal accomplishment (PA). Burnout is costly for physicians and health systems: physicians with burnout experience higher rates of depression, substance abuse, and suicide [3-11], and health systems lose an estimated $4.6 billion annually in direct costs from reduced work hours and physician turnover [12-20]. These estimates exclude the costs of medical errors, malpractice claims, poorer quality of care, and decreased patient satisfaction [21,22].

Organizational strategies to reduce physician burnout, including mindfulness and meditation training, have been described [23]. A systematic review of coaching interventions for physician wellness reported improvement in at least one well-being outcome across the included studies, though the evidence base comprised heterogeneous designs and outcome measures [24]. Coaching has been defined as the activity of bringing forth knowledge, wisdom, and insight through open-ended questions, deep listening, keen observation, self-awareness, and a commitment to learning [25]. Coaching is distinct from mentoring, in which a senior physician shares knowledge and advice with a junior physician [26]; the hierarchical structure of mentoring may limit full disclosure and create an implicit obligation to follow the mentor's advice. Coaching, by contrast, is confidential, and adherence to a code of ethics allows physicians to reflect, reconnect with purpose, and establish goals and action plans for their professional and personal lives [27,28]. Three randomized controlled studies [29-31] reported that professional coaching reduced burnout in primary care physicians [29,30] and surgeons [31], primarily through reductions in EE and/or DP.

All three coaching studies used the validated Maslach Burnout Inventory (MBI) to assess burnout. The MBI has three subscales: EE, DP, and PA. Consistent with the National Academy of Medicine's systems approach to burnout, it is also important to measure the causes of burnout rather than limiting treatment to the individual physician. The Areas of Worklife Survey (AWS) was developed to complement the MBI [32] and assesses six areas (workload, control, reward, community, fairness, and values) that describe which domains of the work environment contribute to physician burnout.

To our knowledge, no prior studies have evaluated physician peer coaching (PPC) in frontline, hospital-based physicians (emergency medicine, hospitalists, critical care, surgeons, medical specialties). Physician peer coaches are physicians who have also completed professional coach training. The primary objective was to determine whether PPC is associated with a greater reduction in MBI EE at six months than no coaching. Secondary and exploratory objectives were to examine associations between PPC and the other MBI subscales, overall burnout (OB), and the six AWS factors. We present the results of a pilot randomized controlled trial testing these questions.

## Materials and methods

Study design, setting, and participants

We conducted a multi-site, single-health-system randomized controlled study with a planned enrollment of 50 hospital-based physicians (emergency medicine, hospitalists, pulmonary/critical care medicine, and other hospital-based specialties) at Geisinger Health System in Pennsylvania, an integrated health system across multiple hospitals. Sample sizes of 25 physicians per group were selected based on a power analysis of 80% power to detect a 6.7-point difference in the MBI EE subscale at a 0.05 significance level (Nsubgroup=23.6) [29]. This calculation was based on EE, which was therefore the primary outcome; the study was not powered for the other outcomes. The study was conducted between June 22, 2022, and June 14, 2023.

Participants were recruited in three tiers; if recruitment in an earlier tier was insufficient, physicians in the next tier were invited. Tier 1 included physicians in emergency medicine, critical care, hospitalists, radiology, and trauma surgery. Tier 2 included physicians in neurology, cardiology, pulmonary medicine, infectious diseases, rheumatology, nephrology, and gastroenterology. Tier 3 included physicians in neurosurgery, orthopaedic surgery, general surgery, thoracic surgery, urology, and vascular surgery. Participants were recruited at departmental meetings, where study team members presented the study and answered questions. Potential participants received an email explaining the study and a link to a Research Electronic Data Capture (REDCap, Vanderbilt University, Nashville, USA) [33] survey to enter demographic and inclusion/exclusion information. Inclusion criteria were that the physician practiced at Geisinger Health System for at least six months. Exclusion criteria were currently seeking professional coaching or planning to leave Geisinger within 12 months. Enrollment proceeded on a first-come, first-served basis until the recruitment goal was met. Informed consent was obtained from all participants. Coaching session content was confidential and was not retained or analyzed outside of this study. The study was approved by the Geisinger Health System institutional review board (IRB approval number: 2022-0144).

Participants were randomized to an intervention group (IG) with six PPC sessions over four to six months or a control group with no PPC. The control group was offered PPC after completing the study. Randomization was performed by the Geisinger Biostatistics Core in REDCap using 1:1 computer-generated block randomization stratified by years of practice and physician specialty.

This trial was registered at ClinicalTrials.gov (NCT05251675) prior to enrollment.

Study groups

Participants randomized to the IG received a one-hour initial coaching session followed by five 45-minute sessions at three- to five-week intervals (4.75 hours of PPC total). All coaching was delivered virtually by a single trained physician peer coach (P.T.), and session attendance was recorded. Coaching topics were unscripted and individualized. The initial session focused on establishing a psychologically safe space and a growth mindset to clarify core values and purpose, identify goals, build self-awareness, and plan professional development. In subsequent sessions, physicians selected their own topics, including debriefing actions taken, assessing ongoing or new challenges, reviewing action plans, strategic planning, and session summary. Participants receiving coaching maintained the same clinical volume as the control group and received no protected time for coaching. Control group participants were encouraged to meet with their existing Geisinger mentor during this period.

The physician peer coach (P.T.) is a co-first author of this manuscript. The coach had no role in outcome data collection, outcome data analysis, or unblinded assessment of survey responses; MBI and AWS surveys were administered and collected by Geisinger Investigator Initiated Research Operations, independent of the coaching relationship, and analyzed by the study biostatistician.

Study outcomes

The primary outcome was burnout, measured by the MBI and its subscales. The secondary outcome was systemic contributors to burnout, measured by the AWS. Baseline, three-month, six-month, and nine-month surveys were administered electronically by Geisinger Investigator Initiated Research Operations. Coaching session attendance was 100%.

Burnout

Burnout was assessed using the Maslach Burnout Inventory-Human Services Survey (MBI-HSS), the most widely used instrument for evaluating physician burnout [29-31,34]. Its psychometric properties are described in the MBI Manual [35], and the instrument is recommended by the National Academy of Medicine for assessing physician burnout [36]. Subjects rate the frequency of 22 items on a 7-point Likert scale from 0 (never) to 6 (daily). Three subscales are reported: EE, DP, and PA. High EE was defined as a score ≥27, and high DP as a score ≥10. We defined high EE as a score ≥27 and high DP as a score ≥10; a subject with high EE and/or high DP was classified as having OB. This composite follows the convention used in prior physician burnout research and the MBI manual thresholds [35]. PA was analyzed as a separate subscale rather than incorporated into the composite because reduced PA emerges later and less consistently than exhaustion and DP and is a less specific marker of burnout.

Work environment

The AWS was developed alongside the MBI to assess work-environment factors contributing to physician burnout [37,38] and has been used previously with physicians [39]. Its six domains are workload, control, reward, community, fairness, and values. Scores reflect the congruence between the physician and the workplace; a score above 3.0 indicates higher congruence, and a score below 3.0 indicates lower congruence.

Statistical analysis

Analysis was performed on a modified intention-to-treat basis: all randomized subjects who received the allocated condition (23 intervention, 22 control) were included, while subjects who withdrew before baseline data collection or otherwise did not receive the allocated condition were excluded. Categorical variables are presented as percentages and continuous variables as medians and interquartile ranges (IQR). Chi-squared tests (categorical variables) and Kruskal-Wallis tests (continuous variables) were used to compare groups at baseline and between groups at specific timepoints. Within-group comparisons of each follow-up time point with baseline used the Wilcoxon signed-rank test for continuous outcomes and chi-squared tests for categorical outcomes. For each group, a repeated-measures model assessed trends across baseline, three, six, and nine months for continuous outcomes, and the Cochran-Armitage trend test was used for categorical outcomes. Within-group trend analyses are exploratory and cannot by themselves establish an intervention effect. Absolute change between baseline and six months was calculated separately for each group, and a difference-in-differences analysis compared the change over time between groups. For continuous subscales, a fixed-effects regression model with repeated measures and compound symmetry covariance was used; estimates, 95% confidence intervals, and p-values are reported. Binary outcomes are reported as odds ratios with 95% confidence intervals from logistic regression. The model included group, time, and group-by-time terms; the group-by-time interaction estimates the between-group difference in change, and the compound-symmetry covariance structure accounts for correlation among repeated measurements within participants. We considered this approach appropriate for a pilot trial of this size, in which the between-group comparison of interest was confined to a single timepoint before control-group crossover. A mixed-effects sensitivity analysis was not performed. The six-month timepoint was chosen for the difference-in-differences analysis because the control group became eligible for coaching at six months, making the nine-month timepoint unsuitable for between-group comparison. Only the primary outcome is considered confirmatory. No adjustment for multiple comparisons was made, and secondary and exploratory results should be interpreted as hypothesis-generating. A p-value less than 0.05 was considered statistically significant. Analyses were performed using SAS, version 9.4 (SAS Institute Inc., Cary, USA).

## Results

Six hundred and fifty physicians were approached. 63 physicians completed REDCap data entry (587 did not complete the eligibility survey; reasons for non-participation were not collected). Participants were enrolled from June 22, 2022, to August 19, 2022. Fifty-one subjects were consented and randomized either to the intervention or the control group (one was excluded because they planned to leave Geisinger within one year; two were excluded because they had not been practicing medicine at Geisinger for ≥ 6 months; nine answered “yes” to seeking professional coaching). Twenty-five subjects were allocated to the IG (two withdrew), and 23 subjects received the allocated intervention. The coaching intervention was conducted from June 24, 2022, to December 14, 2022. Twenty-six subjects were allocated to the control group (one ineligible, one randomization error, two withdrew), and 22 subjects served as the control group. In the IG, 21 subjects completed the surveys at nine months. In the control group, 21 subjects completed the surveys at six months. The study was completed on June 14, 2023, when the last nine-month surveys were completed. Participant flow through recruitment, randomization, and follow-up is reported in Figure 1.

**Figure 1:**
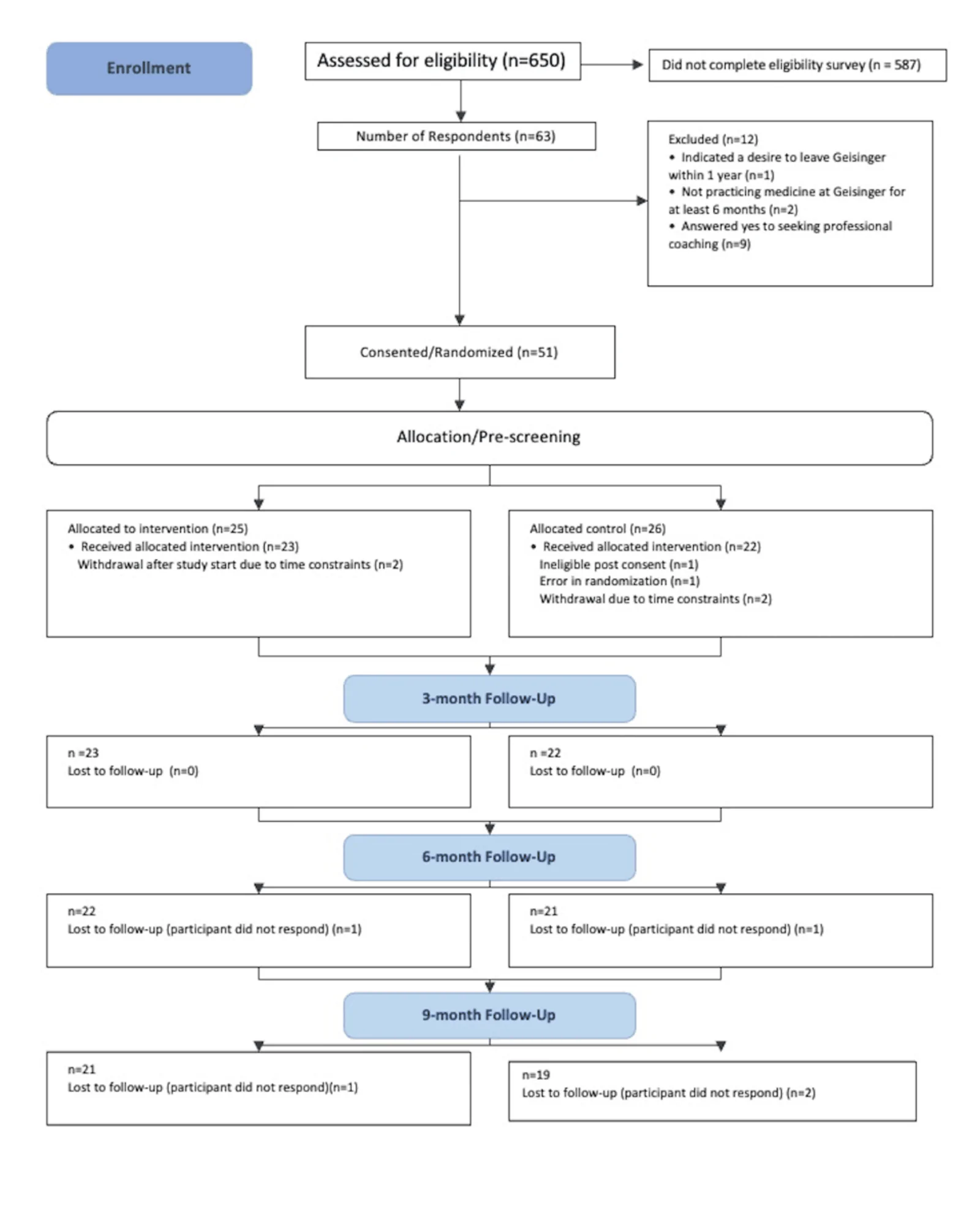
Participant Flow Through Recruitment, Randomization, and Follow-up. Control participants were offered coaching after the 6-month assessment; control-group 9-month data were not used in between-group analyses

The only subject characteristic that had a statistically significant difference between the intervention and control groups at baseline was a higher percentage of males in the control group (47.8% v. 77.3%, p=0.04) (Table 1). No baseline differences were observed in relationship status, location, specialty, years in practice, hours worked per week, proportion of time in direct patient care, or the three MBI subscales and six AWS domains.

**Table 1:** Characteristics of Randomized Groups of the Study. Results are reported as n (%) or median (IQR). 1: Kruskal-Wallis p-value comparing Intervention to Control; 2: Chi-square p-value comparing Intervention to Control; 3: Missing data point. MBI: Maslach Burnout Inventory; EE: emotional exhaustion; DP: depersonalization; PA: personal accomplishment; OB: overall burnout; AWS: Areas of Worklife Survey.

	Intervention (N=23)	Control (N=22)	P-value
Age, years, median (IQR)	38 (34, 45)	37 (35, 46)	0.94^1^
Gender			0.04^2^
Male	11 (47.8%)	17 (77.3%)	
Female	12 (52.2%)	5 (22.7%)	
Relationship status			0.33^2^
Single	1 (4.3%)	4 (18.2%)	
Married	21 (91.3%)	16 (72.7%)	
Divorced/Separated	1 (4.3%)	1 (4.5%)	
Partner	0 (0.0%)	1 (4.5%)	
Location (region)			0.69^2^
Central	8 (34.8%)	9 (40.9%)	
Northeast	14 (60.9%)	11 (50.0%)	
Western	1 (4.3%)	2 (9.1%)	
Specialty			0.95^2^
Emergency Medicine	2 (8.7%)	2 (9.1%)	
Hospital Medicine	10 (43.5%)	8 (36.4%)	
Medical Specialties	5 (21.7%)	7 (31.8%)	
Radiology	2 (8.7%)	2 (9.1%)	
Surgical Specialties	4 (17.4%)	3 (13.6%)	
Number of years in practice			0.94^2^
0-5	11 (47.8%)	9 (40.9%)	
6-10	7 (30.4%)	6 (27.3%)	
11-15	2 (8.7%)	3 (13.6%)	
16-20	1 (4.3%)	2 (9.1%)	
>20	2 (8.7%)	2 (9.1%)	
Number of hours worked per week			0.25^2^
21-40	2 (8.7%)	1 (4.5%)	
41-60	15 (65.2%)	8 (36.4%)	
61-80	4 (17.4%)	10 (45.5%)	
81-100	1 (4.3%)	2 (9.1%)	
>100	1 (4.3%)	1 (4.5%)	
Proportion of time spent in direct patient care			0.20^2^
< 24	1 (4.3%)	1 (4.5%)	
25-49	7 (30.4%)	5 (22.7%)	
50-74	2 (8.7%)	8 (36.4%)	
75-99	9 (39.1%)	7 (31.8%)	
100	4 (17.4%)	1 (4.5%)	
MBI			
EE Total Score	30 (20, 38)	28 (17, 37)	0.63^1^
DP Total Score	6 (3, 10)	7 (4, 13)	0.42^1^
PA Total Score	37 (28, 40)	38.5 (34, 42)	0.43^1^
OB, n (%)	14 (60.9%)	13 (59.1%)	0.90^2^
High EE, n (%)	14 (60.9%)	12 (54.5%)	0.67^2^
High DP, n (%)	7 (30.4%)	8 (36.4%)	0.67^2^
AWS			
Workload Average	2.2 (2.0, 2.8)^ 3^	2.2 (1.8, 3.0)	0.60^1^
Control Average	3.0 (2.3, 3.5)	3.3 (2.5, 4.0)	0.23^1^
Reward Average	3.0 (2.8, 3.5)	3.5 (3.0, 3.8)	0.16^1^
Community Average	3.4 (3.2, 3.6)^ 3^	3.6 (3.4, 3.6)	0.45^1^
Fairness Average	2.8 (2.5, 3.5)	3.1 (2.7, 3.5)	0.40^1^
Values Average	3.5 (3.0, 4.0)	3.8 (3.0, 4.0)	0.69^1^

Between-group comparison at six months

In the IG, EE decreased from a median of 30 (20, 38) at baseline to 18 (12, 35) at six months (p=0.04) and 17 (13, 25) at nine months (p<0.01) (Table 2). High EE decreased from 60.9% at baseline to 31.8% at six months (p=0.05) and 23.8% at nine months (p=0.01). OB decreased from 60.9% at baseline to 40.9% at six months (p=0.18) and 33.3% at nine months (p=0.07), though these individual time-point comparisons did not reach significance. Repeated-measures analyses showed significant trends toward improvement in EE (p<0.01), DP (p=0.01), PA (p=0.02), OB (p=0.05), and high EE (p=0.01); no other burnout measures showed significant trends. Reward scores improved from 3.0 (2.8, 3.5) at baseline to 3.5 (3.5, 3.5) at six months (p=0.04) and 3.5 (3.3, 4.0) at nine months (p=0.01). Repeated-measures analyses showed significant trends toward improvement in workload (p<0.01) and reward (p<0.01); no other AWS domains showed significant trends. Because the control group was offered coaching after six months, the nine-month findings in the IG are uncontrolled. In the control group, no significant decrease in OB was observed from 59.1% at baseline to 52.4% at six months (p=0.66) (Tables 3, 4), and the between-group difference in OB was likewise not significant (Table 5). At six months, the primary outcome, EE, decreased by 6.51 points more in the IG than in the control group (difference-in-differences −6.51; 95% CI −12.70 to −0.31; p=0.04) (Tables 4, 5). Workload congruence, a secondary outcome, improved by 0.57 more in the IG (95% CI 0.13 to 1.01; p=0.01). No other between-group difference reached significance, including OB (OR 0.58; 95% CI 0.17 to 2.05; p=0.40), high EE (OR 0.48; 95% CI 0.15 to 1.52; p=0.21), DP (p=0.78), PA (p=0.14), reward (p=0.14), and the remaining AWS domains. No adverse events occurred.

**Table 2:** MBI, AWS Comparison of 3, 6, 9-months to Baseline for Intervention Group. Responses are reported as Median (IQR) unless otherwise noted. 1: Wilcoxon signed-rank test p-value (paired, within-group comparison with baseline; continuous outcomes); 2: Chi-Square p-value; 3: P-value from repeated measures analysis of trend across Baseline, 3-months, 6-months and 9-months; 4: P-value from Cochran-Armitage Trend Test across Baseline, 3-months, 6-months and 9-months; and 5: Missing data point. MBI: Maslach Burnout Inventory; EE: emotional exhaustion; DP: depersonalization; PA: personal accomplishment; OB: overall burnout; AWS: Areas of Worklife Survey.

		Baseline (N=23)	3-months (N=23)	Baseline v. 3-months P-value		6-months (N=22)	Baseline v. 6-months P-value	9-months (N=21)		Baseline v. 9-months P-value	Trend P-value
MBI											
EE Total Score		30 (20, 38)	25 (17, 33)	0.17^1^		18 (12, 35)	0.04^1^	17 (13, 25)		<0.01^1^	<0.01^3^
DP Total Score		6 (3, 10)	5 (2, 12)	0.83^1^		2.5 (1, 9)	0.15^1^	3 (1, 7)		0.06^1^	0.01^3^
PA Total Score		37 (28, 40)	40 (31, 44)	0.21^1^		41.5 (34, 43)	0.08^1^	39 (35, 43)		0.32^1^	0.02^3^
OB, n (%)		14/23 (60.9)	12/23 (52.2)	0.55^2^		9/22 (40.9)	0.18^2^	7/21 (33.3)		0.07^2^	0.05^4^
High EE, n (%)		14/23 (60.9)	9/23 (39.1)	0.14^2^		7/22 (31.8)	0.05^2^	5/21 (23.8)		0.01^2^	0.01^4^
High DP, n (%)		7/23 (30.4)	7/23 (30.4)	1.00^2^		5/22 (22.7)	0.56^2^	3/21 (14.3)		0.20^2^	0.18^4^
AWS											
Workload Average		2.2^5^ (2.0, 2.8)	2.4 (2.0, 3.4)	0.37^1^		2.4 (2.0, 3.4)	0.24^1^	2.9^5^ (1.9, 3.8)		0.12^1^	<0.01^3^
Control Average		3.0 (2.3, 3.5)	3.3 (2.8, 3.8)	0.41^1^		3.4 (2.5, 3.8)	0.42^1^	3.0 (2.4, 3.8)		0.63^1^	0.49^3^
Reward Average		3.0 (2.8, 3.5)	3.5 (3.3, 3.5)	0.21^1^		3.5 (3.5, 3.5)	0.04^1^	3.5 (3.3, 4.0)		0.01^1^	<0.01^3^
Community Average		3.4^5^ (3.2, 3.6)	3.6 (3.4, 4.0)	0.24^1^		3.7 (3.4, 3.8)	0.07^1^	3.6^5^ (3.4, 3.9)		0.09^1^	0.31^3^
Fairness Average		2.8 (2.5, 3.5)	3.0 (2.7, 3.5)	0.45^1^		3.2 (2.8, 3.7)	0.17^1^	3.0 (2.6, 3.6)		0.45^1^	0.21^3^
Values Average		3.5 (3.0, 4.0)	3.5 (3.0, 4.0)	0.71^1^		3.9 (3.3, 4.0)	0.21^1^	3.6 (3.1, 4.0)		0.55^1^	0.62^3^

**Table 3:** MBI & AWS Comparison of 3- and 6-months to Baseline for Control Group. Responses are reported as Median (IQR) unless otherwise noted. 1: Wilcoxon signed-rank test p-value (paired, within-group comparison with baseline; continuous outcomes); 2: Chi-Square p-value; 3: P-value from repeated measures analysis of trend across Baseline, 3-months, and 6-months; and 4: P-value from Cochran-Armitage Trend Test across Baseline, 3-months, and 6-months. MBI: Maslach Burnout Inventory; EE: emotional exhaustion; DP: depersonalization; PA: personal accomplishment; OB: overall burnout; AWS: Areas of Worklife Survey.

	Baseline (N=22)	3 months (N=22)	Baseline vs 3-months P-value	6 months (N=21)	Baseline vs 6-months P-value	Trend P-value
MBI						
EE Total Score	28 (17, 37)	27.5 (21, 36)	0.96^1^	23 (17, 35)	0.69^1^	0.96^3^
DP Total Score	7 (4, 13)	6.5 (3, 11)	0.52^1^	5 (4, 9)	0.34^1^	0.51^3^
PA Total Score	38.5 (34, 42)	34.5 (30, 42)	0.48^1^	38 (32, 44)	0.89^1^	0.33^3^
OB, n (%)	13/22 (59.1)	13/22 (59.1)	1.00^2^	11/21 (52.4)	0.66^2^	0.65^4^
High EE, n (%)	12/22 (54.5)	11/22 (50.0)	0.76^2^	9/21 (42.9)	0.44^2^	0.44^4^
High DP, n (%)	8/22 (36.4)	7/22 (31.8)	0.75^2^	5/21 (23.8)	0.37^2^	0.37^4^
AWS						
Workload Average	2.2 (1.8, 3.0)	2.2 (1.8, 2.6)	0.45^1^	2.2 (1.8, 2.8)	0.49^1^	0.10^3^
Control Average	3.3 (2.5, 4.0)	3.3 (2.3, 4.0)	0.92^1^	3.3 (3.0, 4.0)	0.51^1^	0.47^3^
Reward Average	3.5 (3.0, 3.8)	3.4 (2.8, 3.8)	0.65^1^	3.5 (3.3, 3.8)	0.79^1^	0.33^3^
Community Average	3.6 (3.4, 3.6)	3.6 (3.2, 3.8)	0.58^1^	3.6 (3.2, 3.6)	0.65^1^	0.46^3^
Fairness Average	3.1 (2.7, 3.5)	3.3 (2.8, 3.5)	0.39^1^	3.2 (2.7, 3.5)	0.88^1^	0.41^3^
Values Average	3.8 (3.0, 4.0)	3.5 (3.0, 4.0)	0.80^1^	3.8 (3.3, 4.0)	0.34^1^	0.50^3^

**Table 4:** MBI & AWS Absolute Change from Baseline to 6-months after Randomization. Responses are reported as Median (IQR) unless otherwise noted.CI: Confidence Interval. 1: Binary outcome; Odds Ratio and respective 95% Confidence Intervals are reported for difference-in-Difference Analysis; 2: Difference-in-differences analysis fixed-effects model with repeated measures p-value; and 3: Missing data point. MBI: Maslach Burnout Inventory; EE: emotional exhaustion; DP: depersonalization; PA: personal accomplishment; OB: overall burnout; AWS: Areas of Worklife Survey.

	Intervention			Control			Difference-in-Difference Analysis	
	Baseline (N=23)(IQR)	6-months (N=22)(IQR)	Absolute Change (IQR)	Baseline (N=22)(IQR)	6-months (N=21)(IQR)	Absolute Change(IQR)	Estimate (CI)	p-value^2^
MBI								
EE Total Score	30 (20, 38)	18 (12, 35)	-5.5 (-9, 3)	28 (17, 37)	23 (17, 35)	2 (-7,5)	-6.51 (-12.70, -0.31)	0.04
DP Total Score	6 (3, 10)	2.5 (1.0, 9)	-2.5 (-4, -1)	7 (4, 13)	5 (4, 9)	-1 (-3,4)	-0.40 (-3.27, 2.47)	0.78
PA Total Score	37 (28, 40)	41.5 (34, 43)	5 (1, 8)	38.5 (34, 42)	38 (32, 44)	1 (-3,5)	2.86 (-1.00, 6.72)	0.14
OB, n (%)	14/23 (60.9)	9/22 (40.9)	-20.0	13/22 (59.1)	11/21 (52.4)	-6.7	0.58^1^ (0.17, 2.05)	0.40
High EE, n (%)	14/23 (60.9)	7/22 (31.8)	-29.1	12/22 (54.5)	9/21 (42.9)	-11.5	0.48^1^ (0.15, 1.52)	0.21
High DP, n (%)	7/23 (30.4)	5/22 (22.7)	-7.7	8/22 (36.4)	5/21 (23.8)	-12.6	1.12^1^ (0.25, 4.99)	0.88
AWS								
Workload Average	2.2^3^ (2.0, 2.8)	2.4 (2.0, 3.4)	0.0^3^ (-0.2, 0.6)	2.2 (1.8, 3.0)	2.2 (1.8, 2.8)	-0.4 (-0.6, 0.2)	0.57 (0.13, 1.01)	0.01
Control Average	3.0 (2.3, 3.5)	3.4 (2.5, 3.8)	0.3 (-0.3, 0.5)	3.3 (2.5, 4.0)	3.3 (3.0, 4.0)	0.0 (-0.3, 0.3)	0.04 (-0.36, 0.43)	0.86
Reward Average	3.0 (2.8, 3.5)	3.5 (3.5, 3.5)	0.3 (0.0, 0.5)	3.5 (3.0, 3.8)	3.5 (3.3, 3.8)	0.0 (-0.3, 0.5)	0.22 (-0.07, 0.51)	0.14
Community Average	3.4^3^ (3.2, 3.6)	3.7 (3.4, 3.8)	0.2^3^ (-0.2, 0.4)	3.6 (3.4, 3.6)	3.6 (3.2, 3.6)	0.0 (-0.2, 0.0)	0.20 (-0.12, 0.53)	0.21
Fairness Average	2.8 (2.5, 3.5)	3.2 (2.8, 3.7)	0.3 (-0.2, 0.3)	3.1 (2.7, 3.5)	3.2 (2.7, 3.5)	-0.2 (-0.2, 0.2)	0.17 (-0.10, 0.44)	0.20
Values Average	3.5 (3.0, 4.0)	3.9 (3.3, 4.0)	0.0 (0.0, 0.3)	3.8 (3.0, 4.0)	3.8 (3.3, 4.0)	0.3 (-0.5 ,0.5)	-0.02 (-0.39, 0.34)	0.89

**Table 5:** Between-Group Difference-in-Differences Estimates, Baseline to 6 Months. Continuous outcomes: difference-in-differences point estimate (intervention change minus control change) from a fixed-effects repeated-measures model with compound-symmetry covariance; binary outcomes: odds ratio from logistic regression (marked OR). Shaded rows reached p<0.05. Only EE was the primary outcome; all others are secondary or exploratory and were not adjusted for multiple comparisons. CI: confidence interval; EE: emotional exhaustion; DP: depersonalization; PA: personal accomplishment; OB: overall burnout; OR: odds ratio; AWS: Areas of Worklife Survey.

Outcome	Difference-in-differences estimate (95% CI)	p-value
EE (primary outcome)	−6.51 (−12.70, −0.31)	0.04
DP	−0.40 (−3.27, 2.47)	0.78
PA	2.86 (−1.00, 6.72)	0.14
OB (OR)	0.58 (0.17, 2.05)	0.4
High EE (OR)	0.48 (0.15, 1.52)	0.21
High DP (OR)	1.12 (0.25, 4.99)	0.88
AWS: Workload	0.57 (0.13, 1.01)	0.01
AWS: Control	0.04 (−0.36, 0.43)	0.86
AWS: Reward	0.22 (−0.07, 0.51)	0.14
AWS: Community	0.20 (−0.12, 0.53)	0.21
AWS: Fairness	0.17 (−0.10, 0.44)	0.2
AWS: Values	−0.02 (−0.39, 0.34)	0.89

## Discussion

This pilot randomized controlled trial suggests that hospital-based physicians who received 4.75 hours of PPC over six months experienced a greater reduction in EE (the primary outcome) and a greater improvement in workload congruence than controls. Within the coaching group, exploratory analyses showed favorable trends in DP, PA, OB, high EE, and reward; however, these were not confirmed by between-group comparison and cannot on their own establish that coaching caused the changes. Improvement across all three MBI subscales within the coaching group differs from previous trials of professional coaching, but this observation requires replication. Prior professional coaching trials demonstrated improvements in EE, high EE, and OB [29,30,40-42] or DP, high DP, and OB [31]. None of these trials demonstrated improvements in PA. This study found the improvements were maintained three months after completion of the coaching intervention. Within-group improvements in the coaching group were still present three months after coaching ended; because the control group was offered coaching after six months, the nine-month data are uncontrolled and cannot be compared between groups.

To our knowledge, this study is the first coaching study to assess change in physicians' perceptions of their work environment. The difference-in-differences analysis showed improvement in workload, and repeated-measures analyses showed significant trends toward improvement in workload and reward. Because no systemic changes to workload or reward structure were made during the study period, improvements in perceived workload may reflect changes in perception or in how physicians organize their work. 

This study included physicians from a range of specialties, including hospitalists, emergency-medicine physicians, critical care, trauma surgery, radiology, and other medical and surgical specialties, consistent with the tiered recruitment scheme described in Methods. It is also among the first coaching studies to collect longitudinal data at four time points (baseline, three, six, and nine months), providing a more complete picture of the intervention's trajectory.

The peer coaching sessions for physicians were designed to be individualized and spontaneous, allowing for personalized discussions. The attendance rate in coaching sessions was 100%. Although a variety of topics emerged, the primary focus was on achieving success in the professional realm. This encompassed areas such as finding fulfillment in work, effectively managing workloads, enhancing efficiency, creating action plans for securing grants and advancing careers, engaging in strategic career planning, cultivating leadership skills, fostering local and national relationships, and achieving work-life balance. The sessions were intended to help physicians connect their purpose and goals with concrete action plans. Alongside these sessions, we observed encouraging and sustained within-group improvements in the measured outcomes. Because multiple MBI and AWS outcomes were examined across several timepoints without formal adjustment for multiple comparisons, the secondary and exploratory results should be interpreted with caution.

A physician who has completed professional coach training [43-48] possesses not only the skills of a professional coach [49-51] but also the experience of navigating the complex healthcare system, which may allow them to understand and empathize with the challenges faced by fellow physicians. In line with recent advice from the US Surgeon General, there was a recommendation to incorporate relationships and social connections into interventions targeting physician burnout [52]. By establishing a psychologically safe space, physician peer coaches can provide support and resources, facilitating open discussions on challenging topics. Peer coaches can also adapt scheduling to physicians' busy and unpredictable clinical schedules; whether these features explain any effect of peer coaching, or improve on external professional coaching, was not tested here.

External professional coaches allow physicians to maintain their clinical duties and generate revenue [29]. However, physician peer coaches may have a more direct understanding of medical practices. The AMA estimates that replacing a physician suffering from burnout costs $500,000 to more than $1,000,000 per physician, including recruitment, sign-on bonuses, lost billings, and onboarding [53]. Given the substantial cost of replacing a physician, the economic value of peer coaching relative to its delivery cost is an important question for future research; however, we did not measure coaching costs, implementation costs, productivity, or quality-adjusted outcomes, and a formal cost-effectiveness evaluation is needed before any economic conclusion can be drawn.

Limitations

This study has several limitations. First, the sample was small (45 analyzed participants) and volunteers; of 650 physicians approached, 587 did not complete the eligibility survey and we collected no information on them, so selection bias cannot be excluded, and generalizability is limited. Second, only the between-group comparison at six months supports inference about coaching; conclusions about other outcomes rest largely on within-group trends, which cannot establish causation, and the nine-month data are uncontrolled because the control group was offered coaching after six months. Third, the primary result is imprecise (95% CI −12.70 to −0.31, with the upper bound close to zero) and should be regarded as preliminary. Fourth, there was no attention-matched control, so the effects of coaching-specific content cannot be separated from nonspecific effects of attention and social contact. Fifth, the analysis was modified intention-to-treat; six randomized participants who did not receive the allocated condition were excluded, which can introduce bias. Sixth, multiple outcomes and time points were examined without adjustment for multiple comparisons, so only the primary outcome is confirmatory. Seventh, randomization resulted in a higher proportion of male physicians in the control group (47.8% vs. 77.3% male, p=0.04); no covariate adjustment was performed because an adjusted model was judged to be underpowered in a pilot of this size, and residual confounding by gender cannot be excluded. Eighth, all outcomes were self-reported; participants and the coach were not blinded, and the coach is an author; although surveys were administered independently and analyzed by the study biostatistician, expectancy and social-desirability bias are possible. Ninth, all coaching was delivered by a single trained physician peer coach; this maximized consistency but prevents assessment of coach-to-coach variability, and whether comparable effects would be achieved by other coaches must be established in a multi-coach trial. Tenth, fidelity was supported only by a common session framework and attendance recording, and session content was not independently assessed. Eleventh, we did not perform a mixed-effects model as a sensitivity analysis; the difference-in-differences repeated-measures model was chosen as appropriate for a pilot, but results might differ under alternative models or missing-data assumptions. Finally, the study was confined to a single health system.

Furthermore, while multiple studies have demonstrated the effectiveness of coaching in reducing physician burnout and improving well-being, the high prevalence of burnout across various specialties highlights the need for broader systemic changes. With burnout rates exceeding 50% in emergency medicine, hospital medicine, family medicine, pediatrics, obstetrics and gynecology, as well as internal medicine, it becomes infeasible to provide coaching to all affected physicians alone. Consequently, there is a pressing need for comprehensive systemic adjustments to standardize workloads and prevent burnout more sustainably.

## Conclusions

This pilot randomized controlled trial suggests that PPC was associated with a significantly greater reduction in EE and a greater improvement in workload congruence than no coaching at six months among hospital-based physicians. Favorable within-group trends in DP, PA, OB, and reward were not confirmed by between-group comparison and should be interpreted as exploratory and hypothesis-generating. Within-group improvements persisted three months after coaching ended, but this observation is uncontrolled. These preliminary findings require confirmation in larger, adequately powered, multi-site trials with more than one trained peer coach, an attention-matched control condition, and formal evaluation of costs.
